# How Well Does ChatGPT-4o Reason? Expert Evaluation of Diagnostic and Therapeutic Performance in Hand Surgery

**DOI:** 10.3390/jcm14228045

**Published:** 2025-11-13

**Authors:** Léna G. Dietrich, Laura De Pellegrin, Valeria Rinaldi, Yves Harder, Esther Vögelin, Esin Rothenfluh

**Affiliations:** 1 Department of Plastic and Hand Surgery, Inselspital University Hospital Bern, University of Bern, 3010 Bern, Switzerland; laura.depellegrin@insel.ch (L.D.P.); valeria.rinaldi@students.unibe.ch (V.R.); esther.voegelin@insel.ch (E.V.); esin.rothenfluh@insel.ch (E.R.); 2 Department of Plastic, Reconstructive and Aesthetic Surgery and Hand Surgery, University Hospital of Lausanne (CHUV), 1011 Lausanne, Switzerland; yves.harder@chuv.ch; 3 Faculty of Biology and Medicine, University of Lausanne, 1011 Lausanne, Switzerland

**Keywords:** artificial intelligence, large language models, ChatGPT-4o, hand surgery, surgical decision-making, diagnostic ambiguity, clinical reasoning, digital health, nerve surgery, medical education

## Abstract

**Background:** The application of large language model (LLM) in surgical decision-making is rapidly expanding, yet its potential in hand and peripheral nerve surgery remains largely unexplored. This study assessed the diagnostic and therapeutic performance of a large language model (ChatGPT-4o) in scenarios characterized by multiple valid management strategies and absent expert consensus. **Methods:** Three representative cases—thumb carpometacarpal (CMC I) arthritis, scaphoid nonunion, and carpal tunnel syndrome (CTS)—were developed to reflect frequent conditions in hand surgery with competing but accepted treatment options. Each case was submitted to ChatGPT-4o using a standardized prompt. LLM-generated responses were evaluated by 52 participants (34 board-certified hand surgeons and 18 residents) across diagnostic accuracy, clinical relevance, and completeness. Readability indices, including Flesch–Kincaid Grade Level, were analyzed to assess appropriateness for a medical audience. **Results:** ChatGPT-4o demonstrated coherent but limited diagnostic accuracy (mean 2.9 ± 1.2 SD), moderate clinical relevance (3.5 ± 1.0 SD), and slightly higher completeness (3.4 ± 1.1 SD). Performance was strongest in the standardized scenario (carpal tunnel syndrome, CTS) and weakest in individualized reasoning (CMC I arthritis). No significant differences were observed between experts and residents (*p* > 0.05). In higher-level reasoning, ChatGPT-4o performed best in CTS and weakest in CMC I arthritis. Readability confirmed professional-level language (mean Flesch–Kincaid Grade Level: 16.4). **Conclusions:** ChatGPT-4o shows promise as a supportive tool for diagnostic reasoning and surgical education, particularly where standardized frameworks exist. Its limitations in ambiguous scenarios highlight the ongoing need for expert oversight. Future large language model development should emphasize specialty-specific training and context-aware reasoning to enhance their role in surgical decision support.

## 1. Introduction

Hand surgery covers a wide range of pathologies that often require highly individualized treatment strategies. Conditions such as flexor tendon injuries [1], degenerative joint diseases [2], post-traumatic complications [3,4], nerve entrapment syndromes [5], and inflammatory arthropathies [6] require precise diagnostic assessment and tailored surgical planning. The complexity of anatomical structures of the hand, combined with the functional significance of even minor impairments, renders decision-making particularly demanding. In clinical practice, these challenges are typically addressed through years of surgical training and experience [7]. However, variability in training exposure, limited case volumes in certain institutions, and the rising complexity of patient profiles can create uncertainty, especially for younger surgeons or in borderline cases where multiple treatment pathways may be viable [8].

Over the past decade, artificial intelligence (AI) has emerged as a potentially transformative tool in the medical field [9,10]. Particularly, large language models (LLMs) have shown promising abilities in synthesizing medical knowledge, generating diagnostic impressions, and formulating potential treatment recommendations [11]. These models are trained on vast datasets, enabling them to provide structured, up to date, and context-aware responses to complex clinical queries. LLM-based tools have already been shown to be useful in supporting diagnostic processes and reducing variability in clinical decision-making in fields such as radiology, dermatology, and internal medicine [9].

However, the application of LLM in surgical specialties remains in its early stages [10]. The nuanced and individualized nature of surgical planning, particularly in hand surgery, represents a challenge to automatization and algorithm-based reasoning. Nevertheless, LLMs’ ability to rapidly analyze clinical information and deliver structured, text-based guidance shows promise for integration into areas such as preoperative planning, patient education, and surgical training [12]. Hence, integration of LLMs into clinical workflows could significantly improve practice and ultimately enhance patient outcomes [13].

Previous studies investigating large language models (LLMs) in surgical contexts have primarily examined their performance in well-defined, algorithmic settings—such as radiology, general surgery, or breast surgery—where diagnostic or therapeutic pathways are standardized and evidence-based consensus exists [12,14]. In contrast, hand surgery frequently involves decision-making in conditions of surgical ambiguity, where several established treatment strategies coexist without a universally accepted “best” option. These scenarios demand nuanced clinical reasoning that integrates patient-specific factors, functional requirements, and surgeon experience. The present study addresses this critical gap by evaluating how ChatGPT-4o, a state-of-the-art LLM, performs when confronted with such ambiguous decision-making situations. Specifically, we sought to assess whether the model can provide coherent and contextually appropriate reasoning when no single correct answer exists, and whether it demonstrates tendencies toward conventional, evidence-based, or innovative approaches when balancing multiple surgical options. By focusing on these inherently uncertain and debate-rich scenarios, this study provides novel insights into how LLMs navigate complexity and variability in surgical reasoning—an essential step toward understanding their potential and limitations in real-world clinical decision support.

## 2. Materials and Methods

This study was designed to evaluate the capacity of ChatGPT-4o (May 2024 version, OpenAI, San Francisco, CA, USA), a state-of-the-art large language model, to support diagnostic reasoning and therapeutic planning in three clinical conditions in the field of hand surgery. The methodological approach was modeled after a previously published study assessing LLM-assisted decision-making in breast surgery and was adapted to the context of upper extremity surgery [12]. The primary objective was to assess the model’s ability to provide accurate, relevant, and complete clinical guidance in response to common surgical scenarios.

The model was asked to provide diagnostic impressions and propose treatment plans based on standardized case vignettes. Its responses were then evaluated by experienced hand surgeons and surgical trainees across three key domains: diagnostic accuracy, clinical relevance, and completeness. This structured evaluation enabled a critical appraisal of how LLM interprets and responds to real-world clinical variability, providing insights into its strengths, limitations, and future potential in surgical decision-making. The findings of this study may contribute to defining the appropriate role of LLM in hand surgery, its integration into surgical training, and its risks of uncritical use. Expert evaluation of ChatGPT-4o may also support the development of future AI tools that better reflect the complexity of clinical practice.

To investigate not only general performance but also how ChatGPT-4o navigates clinical ambiguity, the cases were deliberately selected to reflect situations in which more than one surgical treatment option is considered acceptable and ongoing discussion exists within the surgical community regarding optimal management. These scenarios, where established techniques coexist without clear consensus in the literature or among practicing surgeons, are particularly well suited to assessing how AI models handle real-world decision-making complexity.

Three representative clinical cases were developed to reflect frequently encountered yet diagnostically and therapeutically nuanced conditions in hand and peripheral nerve surgery. All cases were selected by a multidisciplinary team of board-certified hand surgeons to ensure clinical realism, thematic breadth, and relevance to current surgical practice. A key criterion in case selection was the presence of more than one surgically acceptable treatment strategy with ongoing debate regarding the optimal approach. The final selection included: (1) thumb carpometacarpal (CMC I) joint arthritis—a 68-year-old woman with advanced Eaton-Littler stage III disease and functional impairment after failed conservative treatment, requiring evaluation of surgical options; (2) scaphoid nonunion—a 29-year-old athletic male with symptomatic nonunion of the scaphoid following trauma, seeking return to sports and requiring individualized treatment planning; and (3) carpal tunnel syndrome (CTS)—a 54-year-old female with a six-month history of numbness, tingling, and nocturnal symptoms, requiring surgical evaluation and discussion of operative strategies. Each case was designed to simulate a real-world outpatient scenario and included a concise patient vignette, clinical background, and imaging findings [15]. The goal was to challenge the LLMs not only to provide technically correct responses, but also to reveal how it navigates complex decisions in the absence of a single established treatment standard.

A newly created ChatGPT-4o account with no prior conversational history or user-specific customization was used to prevent any bias from previous interactions. All three clinical cases were submitted sequentially within a single session to ensure methodological consistency and to avoid any model fine-tuning effects related to repeated or iterative prompting. Each prompt began with the statement “You are a hand surgeon seeing this patient in consultation”, followed by the detailed case presentation. This framing was chosen to elicit contextually appropriate, clinically grounded responses. The model was instructed to provide a diagnosis, suggest a surgical treatment strategy, and comment on relevant considerations for preoperative planning and postoperative care.

In addition to the core dimensions of diagnostic accuracy, clinical relevance, and completeness, participants also rated three overarching challenge questions for each scenario. These items assessed the clarity of the rationale for preferring one treatment option over another, the balance and clinical relevance of the comparison of options, and agreement with the overall recommendation made in the challenge discussion. Ratings were recorded on the same five-point Likert scale (1 = strongly disagree, 5 = strongly agree) to capture higher-level aspects of reasoning and recommendation quality [16].

The LLM-generated responses were compiled and anonymized for evaluation. The survey was disseminated through the Swiss Society for Hand Surgery (SGH), thereby reaching all registered members of the national professional body. As a result, the survey was accessible to a diverse spectrum of practicing clinicians, including those based in university hospitals, cantonal or regional hospitals, peripheral care centers, and private practices, as well as trainees at various stages of postgraduate education. This distribution ensured broad representation across different clinical settings and levels of experience within the Swiss hand surgery community.

Each participant independently reviewed all three LLM-generated responses and rated them using a five-point Likert scale across three predefined dimensions: diagnostic accuracy (i.e., correctness of the diagnosis based on the information provided), clinical relevance (i.e., appropriateness of the suggested treatment strategies in light of current standards and options), and completeness (i.e., the extent to which relevant clinical aspects were addressed). A score of 1 indicated strong disagreement and 5 indicated strong agreement with the quality of the LLM-generated content [16]. No predefined guideline concordance or case keys were applied; all evaluations were based solely on expert opinion to capture the clinical plausibility and reasoning coherence of the LLM-generated responses. While these single-item Likert ratings provided a practical approach to evaluating the model’s reasoning performance, they do not capture structured trade-off decision-making. This exploratory design was intended to establish a preliminary framework for assessing prioritization tendencies rather than formal preference weighting. More advanced approaches such as discrete choice experiments or pairwise comparisons will be incorporated in future studies to more accurately quantify prioritization skills.

In addition to clinical evaluation, the readability of each LLM response was analyzed using three established readability indices: the Flesch Reading Ease Score (range 0–100, with higher values indicating greater ease of reading), the Flesch–Kincaid Grade Level (corresponding to U.S. school grade levels), and the Coleman–Liau Index (based on character count per word and sentence). These metrics were used to determine whether the LLM-generated texts were appropriately complex for a professional medical audience [17]. Readability was evaluated using the Flesch–Kincaid Grade Level and the Coleman–Liau Index to estimate linguistic complexity and the appropriateness of the text for a professional medical audience.

**Figure 1:**
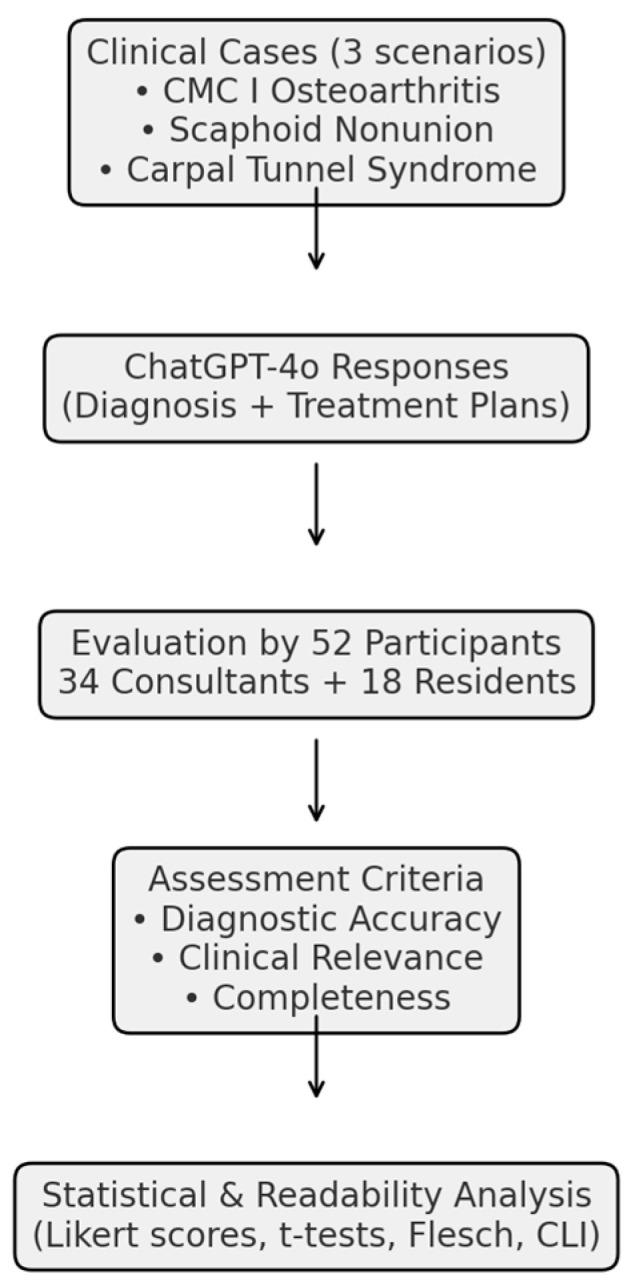
Study workflow for the evaluation of ChatGPT-4o in hand surgery. The study design included three representative clinical cases (CMC I joint arthritis, scaphoid nonunion, and carpal tunnel syndrome), which were submitted to ChatGPT-4o. The LLM-generated diagnostic and therapeutic responses were subsequently evaluated by 52 participants (34 board-certified hand surgeons and 18 residents). Assessments were performed across three domains (diagnostic accuracy, clinical relevance, and completeness), followed by statistical analysis and readability evaluation.

Statistical analyzes were conducted to calculate mean scores across all evaluation dimensions and to compare the responses of attending surgeons and residents using independent-sample *t*-tests [17]. All analyzes were performed using R software, version 4.5.0 (R Foundation for Statistical Computing, Vienna, Austria). Additionally, qualitative comments provided by participants were analyzed thematically to identify common patterns and perceptions related to the model’s clinical reasoning and decision-making behavior, particularly in cases with multiple acceptable treatment paths. As this was an exploratory, descriptive study, no adjustment for multiplicity or hierarchical modeling was applied. Because the ratings were nested (rater × scenario) and the study was exploratory with a limited sample size, no mixed-effects modeling was applied. Analyzes were descriptive, and future studies should employ mixed-effects frameworks to partition rater and scenario variance. Because ratings were collected on 5-point Likert scales, parametric summaries were complemented by nonparametric analyzes. Between-group comparisons (consultants vs. residents) were re-analyzed using Mann–Whitney U tests. Standardized effect sizes (Hedges’ g) with 95% confidence intervals (CIs) were additionally reported. Interrater reliability and within-scenario agreement. Interrater reliability was assessed using the intraclass correlation coefficient ICC(2,1) (two-way random effects, absolute agreement). To account for the ordinal nature of the data, Krippendorff’s α (ordinal) was also calculated. Reliability was summarized (i) across scenarios per domain (pooling the respective items) and (ii) within scenarios for domains containing two or more items. For single-item domains (Completeness), ICC and α could not be computed; we therefore report descriptive dispersion (mean, SD, IQR) and acknowledge this limitation. The methological process is described in Figure 1. 

## 3. Results

### 3.1. Survey Response and Demographics

A total of 300 potential participants, consisting of board-certified hand surgeons and residents in hand surgery were recruited from academic centers and clinical institutions across Switzerland. A total of 52 individuals completed the full evaluation survey. The respondent cohort comprised 34 board-certified Swiss hand surgeons, representing a range of clinical experience and subspecialty expertise, as well as 18 residents currently undergoing formal postgraduate training in hand surgery that lasts at least 4 years. Board-certified hand surgeons (*n* = 34) reported a mean of 8.7 years of professional experience (range 0–34), whereas residents (*n* = 18) reported a mean of 3.1 years (range 0–8). For a comprehensive overview of participant demographics, including distribution by practice setting, years of experience, and training status, please refer to Table 1.

### 3.2. LLM Performance Diagnostic Accuracy, Clinical Relevance, and Completeness Across Clinical Scenarios

Overall, the diagnostic accuracy of ChatGPT-4o showed little variation across the three clinical scenarios, with Likert-scale scores ranging from 2.8 to 2.9 (mean: 2.9). The highest rating was observed for Clinical Scenario 1 (CMC I joint arthritis), with a mean score of 2.9, while the lowest was assigned to Scenario 2 (scaphoid nonunion), which scored 2.8.

In terms of clinical relevance, the average rating across the three scenarios was 3.5. The highest relevance score was observed in Scenario 3 (carpal tunnel syndrome), with a mean of 3.9, followed closely by Scenario 1 (CMC I joint arthritis) at 3.8. The lowest score was reported for Scenario 2 (scaphoid nonunion), which reached 2.7.

Completeness of the LLM responses was more consistent across scenarios. The mean score was 3.4, with Scenario 1 (CMC I joint arthritis) receiving the highest rating (3.4). Scenarios 2 (scaphoid nonunion) and 3 (carpal tunnel syndrome) both scored 3.4, with reviewers noting that although the responses were generally well-structured, they often lacked detailed discussion of surgical nuances.

### 3.3. Comparison Between Groups

**Figure 2:**
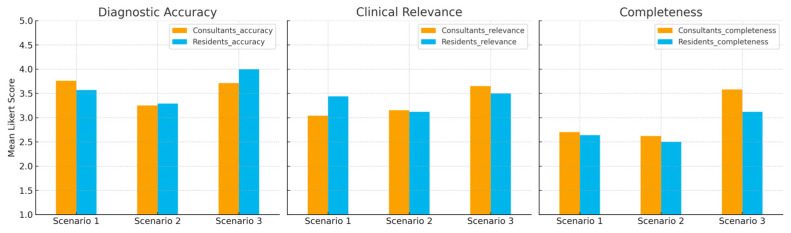
Comparison between consultants and residents. Mean Likert-scale ratings of diagnostic accuracy, clinical relevance, and completeness of ChatGPT-4o responses across three clinical scenarios. No significant differences were observed between board-certified hand surgeons (consultants) and residents.

No statistically significant differences were observed between board-certified surgeons and residents for any of the evaluated dimensions (all *p* > 0.05). This indicates a consistent perception of LLM-generated responses across different levels of clinical experience (Figure 2).

Robustness to ordinal and nonparametric analysis. Across all scenarios and domains, Mann–Whitney U tests revealed no significant differences between consultants and residents (all *p* > 0.30). Effect sizes were small (for example, Scenario 1—Diagnostic accuracy: g = 0.15, 95% CI [−0.51, 0.81]; Scenario 3—Clinical relevance: g = 0.32, 95% CI [−0.41, 1.05]).

Interrater reliability. Reliability was low to moderate across domains (Diagnostic accuracy ICC(2,1) = 0.22, α = 0.12; Clinical relevance ICC(2,1) = 0.11, α = 0.05; Completeness ICC(2,1) = 0.25, α = 0.11). Within-scenario ICCs for Diagnostic accuracy ranged from 0.08 to 0.41 and for Clinical relevance from 0.04 to 0.12. Krippendorff’s α showed a similar pattern. For Completeness within single scenarios, ICC and α could not be estimated.

Contextualizing non-differences (MDD). Given the sample sizes and observed variances, the approximate minimum detectable difference (two-sided α = 0.05, power = 0.80) ranged between approximately 0.8 and 1.2 points on the 5-point scale. Thus, although observed effects were small with CIs including zero, the study was not powered to exclude differences smaller than roughly one scale point.

### 3.4. Readability Analysis

Readability metrics revealed that the LLM-generated responses were generally suited for an audience with medical or advanced academic training. The Flesch Reading Ease scores ranged from 13.3 to 39.2, with a mean score of 22.3, indicating consistently high linguistic complexity across the dataset. The Flesch–Kincaid Grade Level averaged 16.4, aligning with texts typically understood by individuals with graduate-level education. Similarly, the Coleman–Liau Index ranged from 13.7 to 17.7, with a mean of 16.0, further supporting the classification of the content as advanced in readability. These results were consistent across both straightforward and complex clinical scenarios.

### 3.5. Assessment of Challenge Questions

**Figure 3:**
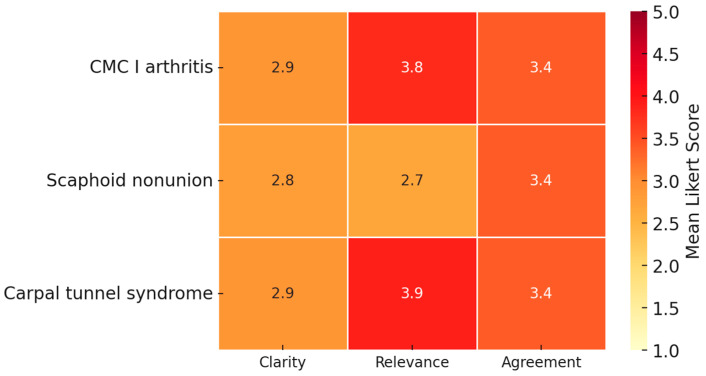
Assessment of challenge questions. Mean Likert-scale ratings for ChatGPT-4o responses to higher-level challenge questions across three clinical scenarios (CMC I arthritis, scaphoid nonunion, and carpal tunnel syndrome). Ratings covered clarity of rationale, balance and clinical relevance, and agreement with the proposed recommendation. The model was perceived as more robust in CMC I arthritis and carpal tunnel syndrome, while less consistent in scaphoid nonunion, a condition characterized by therapeutic ambiguity. Mean ratings ranging from 2.7 to 3.9 indicate mixed perceptions of rationale and balance. These scores should be interpreted descriptively, as no predefined acceptability threshold or external gold standard for prioritization was established.

In the evaluation of the challenge questions, which assessed higher-level reasoning across scenarios, ChatGPT-4o’s performance showed some variation. Results are shown in Figure 3. 

The clarity of the rationale for preferring one treatment over another was rated highest in scenario 1 (CMC I joint arthritis) with a mean score of 2.9 ± 1.2, while scenario 2 (scaphoid nonunion) received the lowest rating (2.8 ± 1.1). Similarly, ratings of the balance and clinical relevance of the comparison of options were most favorable in scenario 3 (carpal tunnel syndrome) (3.9 ± 0.9) and scenario 1 (CMC I joint arthritis) (3.8 ± 1.0), but lowest in scenario 2 (scaphoid nonunion) (2.7 ± 1.1). Agreement with the overall recommendation was highest in Scenario 1 (CMC I joint arthritis, 3.4 ± 1.1) and lowest but nearly identical in scenarios 2 and 3 (both 3.4, SD ≈ 0.9–1.1). These findings indicate that the model’s reasoning and recommendations were perceived as more robust in carpal tunnel syndrome and arthritis, while less consistent in scaphoid nonunion, a case characterized by therapeutic ambiguity. These results are depicted in Table 2. 

### 3.6. Qualitative Feedback

Participants consistently noted the structured format and diagnostic clarity of ChatGPT-4o’s responses. Several evaluators emphasized the utility of the model in delineating standard management approaches and highlighting key elements relevant to clinical decision-making. However, concerns were also raised regarding the model’s limited capacity to tailor its recommendations to individual patient characteristics and anatomical variability. In certain instances, the responses were perceived as overly generic, lacking the depth required to address surgical nuances, patient-specific considerations, and postoperative rehabilitation strategies.

## 4. Discussion

This study evaluated the ability of an advanced large language model, ChatGPT-4o, to provide accurate and clinically relevant recommendations in three representative hand surgical scenarios, deliberately chosen to reflect situations in which multiple, professionally accepted diagnostic or therapeutic strategies exist [18,19,20]. These are the types of cases that frequently generate discussion among hand surgeons, where the absence of a clear consensus requires clinicians to weigh several operative or diagnostic options based on patient-specific factors, surgeon experience, or institutional preferences.

The findings reveal a consistent pattern: ChatGPT-4o demonstrated coherent but limited diagnostic accuracy and a well-structured approach to surgical problems, even in complex or controversial cases. Compared to De Pellegrin et al. [12], who reported higher diagnostic ratings in benign breast conditions undergoing surgical treatment (mean 4.3/5), this study demonstrated lower scores (mean 2.85/5), reflecting the greater therapeutic ambiguity and variability inherent to some type of hand surgery. This contrast underscores the importance of specialty context when evaluating LLM performance in surgical decision-making. Its ability to synthesize clinical information and align it with established diagnostic categories supports its potential as a supplementary diagnostic tool in hand surgery, especially in training settings or environments where immediate expert consultation may not be available. This was particularly evident in scenarios with clear diagnostic features, such as CMC I joint arthritis or carpal tunnel syndrome, where the model provided guideline-conform impressions that mirrored standard clinical reasoning. Across all three scenarios, mean ratings were highest for the carpal tunnel syndrome CTS case, followed by CMC I arthritis and scaphoid nonunion. This observation should be interpreted as a relative finding within the three evaluated scenarios rather than as an absolute indicator of diagnostic or reasoning performance. No human-only or alternative LLM baseline and no predefined content coverage checklist were included to permit external benchmarking.

Surgery of benign breast conditions and hand surgery each present unique diagnostic and therapeutic challenges that resist strict algorithmic approaches. In benign breast surgery, clinical pathways are often supported by established screening protocols and relatively well-defined diagnostic criteria, which may facilitate consensus among experts. In contrast, hand surgery frequently demands nuanced, case-by-case clinical judgment. Variations in injury patterns, patient-reported symptoms, functional demands, and surgeon experience contribute to a broader range of acceptable diagnoses and treatment options—making consensus inherently more difficult to achieve.

The lower Likert scores observed in this study likely reflect both the intrinsic heterogeneity of hand pathologies and the inherent limitations of a text-based LLMs in clinical diagnostics. While this explanation is plausible, it remains speculative, as no predefined case keys or explicit decision criteria were applied to determine whether disagreement coincided with clinically ambiguous areas. Future studies should incorporate structured decision frameworks to differentiate intrinsic variability from model-specific reasoning limitations. Unlike clinicians, ChatGPT-4o cannot integrate crucial sensory information such as visual inspection, palpation, or interpretation of imaging findings—elements that are essential in hand surgery. Therefore, its performance should not be interpreted as diagnostic capability in a clinical sense but rather as an indication of its ability to structure and synthesize medical knowledge within the constraints of textual data. This perspective underscores both the complexity of hand surgical reasoning and the modality-specific boundaries of current LLMs. It suggests that while ChatGPT-4o can organize and interpret clinical information in a manner consistent with standard reasoning patterns, it still lacks the depth and contextual awareness required for more complex or ambiguous diagnostic scenarios. These findings therefore highlight both the model’s internal coherence and its current limitations, reinforcing its potential value as an educational or supportive aid rather than a replacement for expert clinical judgment. However, this interpretation reflects perceived usefulness rather than demonstrated clinical utility. No outcome linkage, user task performance, or diagnostic efficiency metrics were collected in this study. Future work should evaluate whether perceived reasoning quality translates into measurable improvements in clinical decision accuracy or workflow efficiency.

Furthermore, prioritization was assessed through single-item Likert ratings rather than structured trade-off designs, such as discrete choice or pairwise comparison. Consequently, our results provide only an initial descriptive view of how the model ranks competing management options. Future work should employ more formal preference-based methods to evaluate prioritization and trade-off reasoning.

Ultimately, the contrast between De Pellegrin’s study [12] and this study underscores the importance of specialty-specific considerations when assessing LLM performance in surgical decision-making. As both hand and breast surgery are deeply individualized fields, differences in expert ratings may not only highlight variability in LLM interpretation but also reflect the broader spectrum of acceptable clinical reasoning within each domain.

However, the model’s performance was more variable when asked to provide therapeutic recommendations in cases where multiple valid approaches exist. For example, in the scenario of scaphoid nonunion, the LLM-generated responses, while generally reasonable, tended to favor conventional strategies without reflecting the full spectrum of surgical options or clearly articulating the rationale for selecting one over another. This suggests a current limitation in the model’s ability to critically prioritize between competing evidence streams and integrate contemporary surgical debates.

Moreover, ChatGPT-4o frequently lacked the capacity to incorporate nuanced patient-specific considerations, such as occupational demands, hand dominance, or personal treatment goals, which are often decisive in real-world surgical decision-making in hand surgery. This highlights that even when LLM produces guideline-conform outputs, its inability to adapt recommendations to the realities of individual patients currently limits clinical applicability. In practice, the ultimate decision-making authority must therefore remain with experienced surgeons. This limitation is especially important in scenarios where the “best” option cannot be derived from literature alone but requires contextual judgment [21,22]. Accordingly, text-based large language models such as ChatGPT-4o can currently serve only as supportive aids and not as replacements for expert clinical judgment or evidence-based literature. This conclusion is specific to general-purpose LLMs and does not extend to other forms of medical AI, such as domain-trained models or imaging-based classifiers, which have demonstrated substantial diagnostic and predictive potential.

An important methodological consideration in this study is the use of a simplified, standardized prompt format. While the instruction “You are a hand surgeon seeing this patient in consultation” provided consistency and ensured that all case evaluations were framed within a relevant clinical context, it may also have limited the model’s ability to demonstrate its full reasoning potential. Large language models such as ChatGPT-4o are highly sensitive to prompt design, and the structure, specificity, and contextual detail of the input can significantly influence the quality and depth of generated responses. In this study, the use of a single, uniform prompt likely contributed to methodological rigor by reducing variability; however, it may not have fully leveraged the model’s adaptive and interactive capabilities. More advanced prompt engineering—such as iterative questioning, inclusion of additional contextual parameters, or dynamic role specification—could potentially elicit more nuanced, accurate, and clinically sophisticated outputs. Future investigations should therefore explore the impact of prompt design on model performance in medical and surgical decision-making contexts. Our observation was descriptive and not formally tested. Future research should explicitly measure the model’s coverage of the option space, its identification of key discriminators such as patient priorities, bone quality, and occupational demands, and its ability to recommend next diagnostic steps when options are tied.

From a usability perspective, the language complexity of the LLM-generated content was appropriate for a professional audience. Readability scores aligned with graduate-level comprehension, and the responses were generally well-structured and technically accurate [17]. Nevertheless, some participants noted redundancy in phrasing and a lack of stylistic variation, which may reduce clarity or engagement during prolonged use.

Importantly, the study found no significant differences in response ratings between board-certified surgeons and residents, suggesting that the perceived strengths and limitations of the LLMs are recognized consistently across different experience levels. The absence of significant differences between consultants and residents should not be interpreted as equivalence. The effect-size confidence intervals included zero, and the minimum detectable difference (approximately 0.8–1.2 points on the 5-point scale) indicates that smaller effects could not be ruled out. Future studies should predefine equivalence margins and apply formal equivalence testing to determine whether expert groups truly perform comparably.

Interrater agreement was low to moderate, reflecting the variability inherent in subjective expert ratings. Accordingly, we report ICC and Krippendorff’s α and interpret domain-level results with appropriate caution, particularly for single-item domains.

This supports the generalizability of the findings and affirms the potential value of LLM as a uniformly accessible decision support tool.

The analysis of the challenge questions offers further insight into the higher-order reasoning capacities of ChatGPT-4o, particularly in scenarios requiring nuanced clinical judgment. Whereas the model demonstrated generally acceptable performance across a range of clinical contexts, its ability to articulate clear, well-structured rationales and to generate balanced, clinically relevant comparisons was variable and scenario-dependent.

The highest scores were consistently observed in the carpal tunnel syndrome scenario, a condition with well-established diagnostic criteria and standardized management pathways. In this context, the model was able to present coherent therapeutic strategies, supported by current evidence and aligned with prevailing clinical guidelines, resulting in outputs that were rated as both accurate and educationally valuable.

In contrast, the lowest ratings were reported in the scenario involving CMC I joint arthritis. This condition is characterized by a broad spectrum of surgically acceptable interventions, ranging from trapeziectomy and ligament reconstruction to arthroplasty and fusion, with ongoing debate in the surgical community regarding optimal management. In this setting, the model’s responses were frequently perceived as insufficiently differentiated. While the LLM reliably identified standard treatment options, it tended to default to conventional strategies without adequately exploring the range of alternatives, the clinical factors influencing their selection, or the evidence-based trade-offs between them. Moreover, the responses often lacked a critical appraisal of current controversies, failing to contextualize therapeutic choices within the evolving landscape of surgical practice.

These findings highlight a key limitation of current large language models: while they are proficient at replicating structured diagnostic reasoning and summarizing common therapeutic approaches, they exhibit reduced reliability in situations requiring prioritization among competing strategies, particularly where evidence is equivocal or expert consensus is lacking. In such scenarios, the model’s reasoning remains largely descriptive rather than analytical, limiting its utility for advanced clinical decision-making.

Accordingly, while ChatGPT-4o may serve as a supportive tool in synthesizing established knowledge and guiding learners through routine clinical cases, its application in complex or controversial decision-making underscores the ongoing need for expert human judgment and multidisciplinary discussion. This is particularly critical in fields such as hand surgery, where treatment often requires careful balancing of patient-specific factors, surgeon experience, and evolving clinical evidence [23].

Overall, the results are consistent with growing literature positioning large language models as assistive tools rather than independent decision-makers in surgical specialties [12,24,25,26,27]. In hand surgery specifically, such tools may provide educational value for trainees, offer structured guidance in low-resource settings without subspecialty expertise, and support patient education through consistent baseline information. These application domains could accelerate safe integration of LLM into clinical workflows even before model performance reaches expert level. While ChatGPT-4o demonstrates substantial potential to support clinical reasoning and provide educational scaffolding in hand surgery, its limitations, particularly in the context of non-standardized, expert-level decisions, reinforce the necessity of human oversight. The ability to navigate uncertainty, integrate contextual variables, and balance competing strategies remains a distinctly human skill.

Beyond their potential in clinical decision support, LLM-generated responses may also provide educational value for surgical trainees and offer accessible guidance in low-resource settings where subspecialty expertise is not readily available [13].

Limitations of this study include the relatively small sample size, the restriction to Swiss participants, and the lack of evaluation against real patient outcomes.

The selection of evaluators was limited to hand surgeons and residents in hand surgery practicing in Switzerland, reflecting the study’s design for potential distribution through the Swiss Society for Hand Surgery. While this approach ensured homogeneity in training background and clinical context, it may limit the generalizability of the findings to international settings. Although this restriction does not introduce bias in the interpretation of the survey results, it represents a potential limitation. Future studies should therefore aim to include a more geographically diverse group of evaluators to capture potential regional or cultural variations in clinical reasoning and assessment.

Finally, readability metrics such as the Flesch–Kincaid and Coleman–Liau scores provide insights into linguistic complexity rather than factual density or clinical calibration. These indices were applied to characterize text accessibility and not to evaluate diagnostic validity or reasoning accuracy. Readability was assessed at the aggregate level without per-scenario variance or correlation analyzes; future work should explore how linguistic readability relates to expert evaluation metrics

Furthermore, only three representative cases were evaluated, which limits the generalizability to the full spectrum of hand surgical conditions These conditions, CMC I arthritis, scaphoid nonunion, and carpal tunnel syndrome, were selected to encompass chronic degenerative, post-traumatic, and compressive pathologies, thereby capturing different levels of surgical ambiguity. Expanding the number of cases could have improved generalizability but would have substantially increased the time investment required of participants, likely further reducing the response rate. ChatGPT was selected as the sole model for evaluation, as it currently represents the most widely recognized and commonly used large language model among healthcare professionals, including within our institution. This ensured realistic assessment conditions and participant familiarity while avoiding the conceptual and time-related burden of comparing multiple models within a single survey. Although this approach limits cross-model generalizability, it enhances methodological clarity and provides a focused foundation for future comparative and multi-center investigations. [28].

In summary, ChatGPT-4o demonstrates potential as an adjunctive tool for diagnostic reasoning and foundational treatment planning in hand surgery, particularly in scenarios governed by well-defined diagnostic criteria and standardized management protocols. Its applicability remains more limited in conditions characterized by therapeutic ambiguity, where individualized clinical judgment is essential. Moreover, its current capabilities are restricted in complex or controversial contexts that demand nuanced reasoning and tailored decision-making. Future improvements, such as the incorporation of specialty-specific knowledge, context-sensitive training methodologies, and continuous integration of evolving clinical guidelines and debates, may enhance the model’s applicability and reliability in real-world surgical practice. Until such advancements are realized, expert clinical judgment remains indispensable, particularly in areas where therapeutic decisions are shaped by ongoing professional discourse and the absence of definitive consensus.

## 5. Conclusions

ChatGPT-4o demonstrated coherent but limited diagnostic accuracy and clinically relevant responses in representative hand and peripheral nerve surgery scenarios where multiple valid strategies exist but no consensus is clear. In CTS, it consistently recognized established diagnostic criteria and recommended standard decompression, making it a potentially valuable aid for trainees or in non-specialist settings. In CMC I arthritis, it reliably outlined operative options such as trapeziectomy, ligament reconstruction, and arthroplasty, thereby structuring the decision-making landscape even though it could not prioritize between techniques. In scaphoid nonunion, it identified conventional strategies, including bone grafting and fixation, but lacked adaptability to account for patient-specific demands such as athletic activity. Overall, the model offered structured, guideline-conforming recommendations but lacked nuance and individualization, tending toward conventional approaches rather than engaging with ongoing debates or tailoring to individual patients. These findings suggest that large language models can support education, training, and standardized preoperative planning, but their limitations highlight the need for cautious, supervised use and underscore the irreplaceable role of human expertise in ambiguous cases. Future model development should emphasize specialty-specific training, integrating surgical literature, expert case discussions, and operative data, and improved context-aware reasoning to account for patient- and setting-specific variables. These refinements will be essential to advance large language models from general text generators toward reliable, specialty-adapted assistants in surgical decision-making.

## Figures and Tables

**Table 1:** Participant Demographics.

Characteristic	Value
Total evaluations	52
Board-certified hand surgeons	34
Residents (plastic or hand surgery)	18
Years since board certification (mean ± SD)	8.7 ± 8.7
Years of surgical training (residents, mean ± SD)	3.1 ± 1.0

**Table 2:** Mean Likert Scores (± SD) by Scenario.

Scenario	Diagnostic Accuracy	Clinical Relevance	Completeness
CMC I Joint Arthritis	2.9 ± 1.2	3.8 ± 1.0	3.4 ± 1.1
Scaphoid Nonunion	2.8 ± 1.1	2.7 ± 1.1	3.4 ± 0.9
Carpal Tunnel Syndrome	2.8 ± 1.1	3.9 ± 0.9	3.4 ± 1.1

## Data Availability

The original contributions presented in this study are included in the article. Further inquiries can be directed to the corresponding author.
